# Reviewed article: Pereira, MIM; et al. American tegumentary leishmaniasis: epidemiological analysis and time series, Minas Gerais, Brazil, 2008-2024. Epidemiol Serv Saude. 2026:35;e20251212

**DOI:** 10.1590/S2237-96222026v35e20251212.c

**Published:** 2026-11-09

**Authors:** Max Moura de Oliveira

**Affiliations:** 1 Universidade Federal de Goiás, Goiânia, Goiás/GO, Brazil. Universidade Federal de Goiás Goiânia GO Brazil

## First round comments

Prezados Autores,

Agradecemos o interesse em publicar na Epidemiologia e Serviços de Saúde (RESS) o manuscrito “Leishmaniose tegumentar americana no norte de Minas Gerais: análise epidemiológica e série temporal (2008-2024)”. Após a avaliação do manuscrito pelos revisores, concluímos que o estudo aborda um tema de relevância para a vigilância em saúde em Minas Gerais. No entanto, identificamos fragilidades metodológicas e estruturais que impedem sua aceitação no formato atual.

A decisão editorial é de REVISÃO MAIOR, condicionada a uma reestruturação obrigatória do manuscrito para a categoria de NOTA DE PESQUISA.

Entendemos que os achados do estudo configuram um diagnóstico situacional importante, mas não apresentam justificativa, como densidade analítica ou a complexidade de desenho requerida para um Artigo Original completo na RESS. O formato de Nota de Pesquisa valorizará a objetividade dos seus dados, permitindo uma comunicação mais focada.

Assim, é importante que vocês atendam aos seguintes pontos, que se sobrepõem a eventuais sugestões divergentes dos revisores:

1. Adequação de Formato: Ajustem o texto rigorosamente às normas de “Nota de Pesquisa” (limite de palavras, tabelas e referências), conforme instruções disponíveis em: https://ress.iec.gov.br/p/page/2/instrucoes. Inclusive, reorganizar as três tabelas em uma única tabela.
2. Correção Metodológica (Fonte de Dados): Há uma contradição na descrição dos métodos. O texto cita uso de dados tabulados (TabNet), mas menciona procedimentos de “limpeza de duplicatas” incompatíveis com dados agregados. Esclareçam a fonte real ou corrijam a descrição do processamento.
3. Revisão Estatística: O resultado do teste de Mann-Kendall apresentado (p=1,000) sugere um possível erro de cálculo ou artefato estatístico. A análise de tendência deve ser refeita e verificada antes de qualquer interpretação.
4. Foco da Discussão: Evitem expandir o texto com revisões bibliográficas básicas sobre o vetor ou a doença (biologia, sintomas gerais). Foquem a discussão nas falhas das medidas de controle diante da estabilidade da endemia e nas características regionais (autoctonia e formas clínicas), conforme apontado pelos revisores.

Solicitamos que enviem, juntamente com a nova versão, uma carta resposta detalhando como cada ponto foi atendido.

Atenciosamente,

Max Moura de Oliveira

Editor Associado

Epidemiologia e Serviços de Saúde

COMENTÁRIOS DOS REVISORES (Consolidados pelo Editor)

A seguir, apresentamos as recomendações dos revisores agrupadas por nível de prioridade para facilitar sua revisão. Quando possível, os comentários foram ordenados seguindo a estrutura lógica do manuscrito (seções indicadas entre parênteses). Ressaltamos que o texto dos pareceres foi mantido na íntegra, apenas reordenado.

A adesão aos itens “Críticos” e “Importantes” é fundamental para a continuidade do processo editorial, entretanto, todas as recomendações listadas abaixo devem ser respondidas ponto a ponto na carta resposta.

REVISOR 1

Comentários importantes (Correções maiores)

(Métodos) “Inserir após o Delineamento do estudo um tópico de Contexto. Neste tópico contexto escrever sobre as dimensões e população do Norte de Minas e citar as macrorregiões ou cidades do norte de Minas. Qualquer dúvida, verificar em artigos já publicados na RESS. Segue o link de um exemplo de artigo: [Link omitido - verificar PDF original se necessário]”

(Variáveis) “Por que a categoria ‘ignorado/branco’ foi excluída de todas as variáveis? Essa categoria é importante para verificar a incompletude dos dados. Além disso, implica na análise das tendências de incidência e na identificação de padrões de persistência e vulnerabilidade. Assim, podería destacar na discussão o quão importante é preencher a ficha de notificação da doença corretamente pelo profissional de saúde.”

(Resultados/Tabelas) “O que significam os números na tabela 1? Seria algum código dos municípios? Se sim, por favor inserir uma coluna na tabela com o nome código do município.”

(Resultados) “Agrupar as faixas etárias em menos categorias para facilitar a compreensão do leitor”

(Discussão) “Faltam dados de estudos atuais no Brasil e no mundo sobre a Leishmaniose tegumentar americana. A discussão precisa ser mais ampla. Comparar os resultados do trabalho com outros estudos em outras regiões do país ou até mesmo de outras regiões de Minas Gerais.”

(Discussão) “Utilizar dados internacionais de países endêmico e utilizar estudos atuais.”

(Discussão) “Discutir melhor a implementação de políticas públicas para combater/prevenir a doença ou os flebotomineos.”

(Discussão) “Melhorar o último parágrafo da discussão de uma forma que esclareça a importância de desenvolvimento econômico e social, e políticas públicas que ajudem a combater as regiões endêmicas.”

Comentários desejáveis (Correções menores)

(Título) “Sugestão de título: Leishmaniose tegumentar americana no norte de Minas Gerais: análise epidemiológica e série temporal, 2008-2024”

(Aspectos éticos) “Utilizar a frase padrão da revista que é: Esta pesquisa utilizou bancos de dados de domínio público e anonimizados.”

(Aspectos éticos) “Retirar os aspectos éticos antes do resultado. É NECESSÁRIO APENAS O ASPECTOS ÉTICOS DEPOIS DO RESUMO.”

(Introdução) “Primeiro parágrafo rever a seguinte frase: sendo apresentada com ampla distribuição no Brasil e no território brasileiro, configurando-se como um relevante desafio para a saúde pública e elevada carga de morbidade. Existe uma redundância na frase. Escrever de forma mais completa e simples.”

(Variáveis) “Citar apenas as variáveis da seguinte forma: raça/cor da pele, sexo, faixa etária e região de residência.”

Comentários desejáveis (Correções menores - caso haja palavras disponíveis)

(Introdução) “Inserir na introdução mais informações sobre o protozoário do gênero Leishmania.”

(Introdução) “Fazer uma breve explicação sobre os flebotomíneos com o propósito de enriquecer o texto com mais informações.”

(Introdução) “Inserir também mais sintomas, diagnóstico e tratamento da doença.”

REVISOR 2

Comentários importantes (Correções maiores)

(Introdução/Justificativa) “Justificativa do estudo precisa ser mais objetiva. Embora a relevância esteja implícita, falta explicitar claramente o diferencial do trabalho. Explorar mais a importância e porque de: série temporal longa; importância para vigilância epidemiológica regional. Sugiro reforçar esse argumento ao final da Introdução.”

(Métodos) “Métodos carecem de pequenas explicitações que fortalecem rigor científico”

(Métodos) “Linhas 62-65: explicar como casos duplicados/inconsistentes foram identificados.”

(Métodos) “Linha 96: justificar exclusão da categoria ‘ignorado/branco’.”

(Métodos) “Linha 86: especificar o pacote do R utilizado para o teste de Mann-Kendall.”

(Métodos) “- Variáveis de faixa etária muito segmentadas. Essa categorização foi baseada em que? Um estudo? Algum documento técnico?”

(Métodos) “Linha 88: indicar data exata de download da base.”

(Resultados) “Linha 108: incluir as taxas anuais mínimas e máximas.”

(Resultados) “Linha 119: comentar proporção relativamente alta de ‘indeterminado’ (5,9%). E levar para discussão para ampliar os argumentos.”

(Discussão) “Discussão pode ser mais crítica e menos descritiva. Em alguns trechos, há repetição de informações dos Resultados sem aprofundar implicações epidemiológicas. Nesse sentido, deixo como sugestão: integrar comparação com tendências nacionais e estaduais; explorar implicações para vigilância, diagnóstico e controle vetorial; refletir sobre impacto de autóctones elevados; incorporar limitações do estudo (dados secundários, subnotificação, ausência de análise espacial).”

(Discussão) “Linhas 162-165: esclarecer que o estudo não realizou análise espacial, embora dialogue com literatura que fez.”

(Discussão) “- Linhas 180-187: aprofundar discussão sobre formas mucosas e diagnóstico.”

Comentários desejáveis (Correções menores)

(Introdução) “A Introdução deve ser revisada integralmente para melhorar fluidez, precisão e correção gramatical. Trechos com estrutura inadequada, redundâncias, falta de verbos principais e repetições.”

(Introdução) “Linhas 14-16: frase incoerente e redundante (‘...ampla distribuição no Brasil e no território brasileiro...’). Sugiro reescrita completa.”

(Introdução) “Linha 19: ausência de conjunção (‘mantém caráter endêmico está associada’).”

(Introdução) “Linhas 17-20: falta de pontuação; inserir ‘e’: ‘mantém caráter endêmico e está associada...’.”

(Introdução) “Linhas 20-27: ideias repetidas sobre urbanização, ecossistemas e desigualdade.”

(Introdução) “Linhas 31-36: duplicação do argumento sobre flutuações temporais.”

(Introdução) “Linhas 38-46: frase longa com excesso de vírgulas; sugerir divisão em duas sentenças.”

(Discussão) “- Linhas 150-154: repetição do argumento sobre irregularidade temporal.”

(Redação Geral) “Quanto à redação geral do manuscrito, deixo como observações: Evitar repetição do termo ‘macrorregião norte de Minas Gerais’ quando o contexto já estiver claro. Simplificar frases extensas. Padronizar termos epidemiológicos.”

REVISOR 3

Segue parecer relativo ao artigo “Leishmaniose tegumentar americana no norte de Minas Gerais: análise epidemiológica e série temporal (2008-2024)”.

Um estudo importante que cobre um período extenso de 17 anos (2008-2024), permitindo uma visão da evolução da doença. A amostra de 7.275 casos confirmados é expressiva e confere solidez aos achados epidemiológicos

Por outro lado, é importante notar que estudos epidemiológicos descritivos baseados em dados secundários (SINAN) e análises de séries temporais são amplamente utilizados na literatura científica brasileira para o monitoramento de doenças negligenciadas. Esse formato é bastante utilizado para a vigilância em saúde, pois permite que gestores identifiquem o perfil da população acometida e áreas de risco. No entanto, por ser um modelo comum, o valor do artigo reside menos na inovação metodológica e mais na sua extensa abrangência temporal (17 anos).

Embora o artigo apresente resultados sólidos, segue alguns pontos na discussão que podem ser explorados com maior profundidade:

Comentários importantes (Correções maiores)

(Discussão) “Determinantes da Alta Autoctonia (90,8%): O fato de quase 91% dos casos serem autóctones, a discussão poderia expandir a análise sobre como a urbanização desordenada e a modificação de ecossistemas locais no norte de Minas estão permitindo que o ciclo de transmissão se mantenha de forma tão eficiente dentro dos municípios, sem depender de casos importados.”

(Discussão) “Impacto da Forma Mucosa (6,6%): As fontes indicam que a forma mucosa está associada a diagnósticos tardios ou falhas no tratamento anterior. Uma discussão mais profunda poderia investigar se a persistência dessa forma clínica em 482 casos decorre da circulação específica da Leishmania (Viannia) braziliensis na região ou se é um reflexo direto da precariedade no acesso à saúde por populações vulneráveis.”

(Discussão) “Vulnerabilidade Étnico-Social: O estudo aponta que pessoas pardas e indígenas representam a maioria dos casos, sugerindo barreiras de acesso ao sistema de saúde. A discussão ganharia mais relevância se explorasse as barreiras culturais e geográficas específicas que essas populações enfrentam, indo além da simples constatação estatística.”

(Discussão) “Análise da ‘Estabilidade’ Temporal: O teste de Mann-Kendall revelou uma tendência estacionária (p-valor=1,000). Isso significa que, em quase duas décadas, as políticas de controle não foram capazes de reduzir a incidência. O texto poderia discutir de forma mais crítica porque as estratégias de vigilância e controle vetorial parecem estar estagnadas ou ineficazes para alterar a curva epidemiológica na região.”

(Resumo/Conclusão) “Em resumo, o artigo cumpre com rigor o seu papel de diagnóstico regional, mas a discussão poderia ser o espaço para uma reflexão mais provocativa sobre o fracasso das medidas de controle convencionais diante de uma doença que se mantém estável e resiliente no norte de Minas Gerais há 17 anos.”

REVISOR 4

Comentários críticos (Correções prioritárias)

(Integridade/Similaridade) “1. Remover similaridades identificadas no ‘Relatório iThenticate’ anexo, em especial sentenças completas. Após eventual aceite do manuscrito, o limite de 30% precisa ser assegurado para indexação. No entanto, mesmo que esteja dentro do limite, orientamos que revise o texto para assegurar a originalidade do manuscrito a partir dos trechos identificados no relatório em anexo, se pertinente.”

(Descritores) “2. Inserir 5, selecionadas a partir da lista Descritores em Ciências da Saúde (DeCS...). No seu caso, há 2 termos que não estão exatamente da mesma forma que o DECs publica, portanto, é preciso verificar e corrigir. Apresentar primeira letra de cada palavra em maiúscula, conforme os DeCS, separadas por ponto e vírgula; incluir descritor referente ao desenho do estudo como último termo.”

(Objetivos) “3. O objetivo listado no resumo difere do escrito no último parágrafo da Introdução.”

Comentários desejáveis (Correções menores)

(Aspectos éticos) “4. Nos Métodos, remover o tópico sobre ‘aspectos éticos’, pois as informações já devem constar apenas no quadro de aspectos éticos.”

(Formatação) “5. Após siglas como ‘APC’, ‘VPA’, ‘VPMA’, ‘RP’... bem como após p-valor, não usar sinal de igual (=) ou dois pontos (:) entre a sigla/termo e o número, deixar apenas um espaço. Esta orientação também vale para tabelas e figuras.”

(Formatação) “6. Utilize hífen (‘-’) para intervalos numéricos das categorias (ex.: 0-4) e assegure compatibilidade entre as categorizações apresentadas nas tabelas e aquelas informadas nos métodos, com consistência em todo o texto.”

(Referências) “7. Revisar as referências. [...] Além de observar o estilo de citação e referenciamento, os autores devem observar critérios para seleção das referências. As referências citadas indicam atualização e conexão com investigações relevantes dos autores e seu texto. Recomenda-se citar pesquisas científicas relevantes... A boa prática na revisão da literatura e construção do texto veta a citação de citação (apud)... Tal procedimento configura citação indireta e introduz erros factuais no texto.”

REVISOR 5

Comentários críticos (Correções prioritárias)

(Delineamento) “3- Delineamento do estudo: denominar o tipo de estudo de forma direta, não inserir termos redundantes (ex.: estudo epidemiológico, observacional...). Sugere-se denominar somente ‘estudo descritivo’. Ajustar o título, resumo e seção de métodos do texto principal;”

(Resumo) “4- Resumo (métodos): inserir os tipos de medidas calculadas e organizar as informações das seções da seguinte forma: tipo de delineamento, fonte das informações utilizadas e tipos de medidas calculadas;”

(Resumo) “6- Resumo (resultados): não repetir informações metodológicas (teste de Mann-Kendall) nos resultados. Evitar utilizar ‘significativo’, apresentar somente os resultados de forma clara. Rever essa frase (...) e utilizar uma linguagem simples para que o leitor tenha fácil interpretação ao ler.”

(Resumo) “7-Resumo (conclusão): deter-se ao que se pode afirmar a partir dos resultados apresentados no resumo em resposta ao objetivo.”

(Tabelas) “11- Tabela 1: explicar os números/códigos inseridos antes do nome dos municípios. Refletir sobre a importância de mantê-los na tabela e se decidir manter, identificar adequadamente no cabeçalho;”

(Tabelas) “12- Tabela 2: ajustar o cabeçalho para: I Variáveis I n (%) I”

(Figuras) “13- Figura 1: ajustar a legenda. Aumentar um pouco o tamanho dos números e utilizar somente um hífen nos intervalos numéricos da legenda (ex.: 188-253). Não é necessário inserir ‘LEGENDA:’, remover;”

(Figuras) “14- Figura 2: rever o título e remover a frase sobre os pontos e a linha azul (‘ Os pontos indicam valores anuais e a linha azul representa tendência suavizada da série temporal’). Sugere-se inserir uma legenda;”

(Figuras) “15- Figura 2: rever o eixo x, os anos estão muito próximos e confusos. Inserir um espaço entre os anos para ficar mais clara a visualização;”

(Tabelas) “16- Tabelas: Alinhe os dados numéricos à direita e os de texto à esquerda nas células”

(Tabelas) “17- Tabelas: O título, a tabela e suas notas devem caber em duas páginas A4... A fonte padrão é Times New Roman... Utilize a opção ‘Repetir linhas de cabeçalho’ do Word... Linhas e colunas de tabelas devem ser criadas com recurso de tabela, sem uso de espaço ou quebra de parágrafos.”

(Tabelas/Figuras) “18- Títulos de ilustrações: rever e ajustar; devem ser claros e informar o conteúdo da ilustração... títulos devem ser autossuficientes... Separar termos por vírgula e não incluir ponto no final. Siglas essenciais para compreensão da ilustração, devem constar preferencialmente no título...”

Comentários importantes (Correções maiores)

(Título) “1- Título do manuscrito: informar o tema principal, delineamento, local e ano(s) da pesquisa. Não utilizar parênteses no título;”

(Métodos - STROBE) “8- Seção de métodos (texto principal): rever. Recomenda-se aderir consistentemente ao checklist STROBE para relato de estudos observacionais. A seção de métodos deve ser ajustada e as informações devem ser desmembradas e completadas de acordo com os seguintes itens: Delineamento do estudo; Contexto; Participantes; Variáveis; Fontes de dados/ mensuração; Viés; Tamanho do estudo; Métodos estatísticos. Acesse: https://www.strobe-statement.org/”

(Discussão) “10- Discussão: rever cuidadosamente, não repetir resultados e seguir as instruções: o primeiro parágrafo da discussão deve resumir os achados, interpretando-os e sem repetir números nem citar ilustrações ou referências... Apresentar as limitações do estudo... Após esclarecer as limitações da pesquisa, o texto da discussão deve ser guiado pelos seus achados... Assegurar que a discussão faz a ponte entre os achados e pesquisas compatíveis... O último parágrafo (curto e conciso) deve apresentar a conclusão da pesquisa com base do que se pode afirmar a partir dos resultados.”

Comentários desejáveis (Correções menores)

(Resumo) “2- Resumo (objetivo): Utilizar hífen para intervalos de data (ex: 2008-2024). Ajustar as outras menções semelhantes no texto principal e ilustrações;”

(Resumo) “5- Resumo (resultados): utilizar hífen para intervalos numéricos. Ajustar a faixa etária para ‘20-59 anos’;”

(Métodos) “9- Seção de métodos (texto principal): remover o item ‘aspectos éticos’. Essas informações devem ser inseridas somente no quadro abaixo do resumo;”

(Referências) “19- A referência 19 não consta no texto. Rever e esclarecer;”

Recommendation: Major revision 

## Second round

Prezadas(os) autoras(es),

Agradecemos o interesse pela Epidemiologia e Serviços de Saúde (RESS) e parabenizamos pelo avanço substancial no atendimento às sugestões. A R1 apresenta melhorias claras de estrutura (Nota de Pesquisa), foco da discussão e aderência ao STROBE. Entretanto, alguns pontos não foram completamente esclarecidos ou não identificamos mudanças substantivas no arquivo, motivo pelo qual recomendamos revisão adicional e/ou justificativa objetiva ponto a ponto. Por fim, registramos inconsistências internas observadas na R1 que precisam ser corrigidas antes do reenvio.

Abaixo organizamos: (1) itens ainda pendentes do primeiro parecer (com as recomendações enviadas anteriormente e as respectivas fontes); e (2) novas recomendações editoriais de clareza/redação com base na R1.

Itens pendentes do primeiro parecer

- Comentários importantes (correções maiores)

“3. Revisão Estatística: O resultado do teste de Mann-Kendall apresentado (p=1,000) sugere um possível erro de cálculo ou artefato estatístico. A análise de tendência deve ser refeita e verificada antes de qualquer interpretação.” [Editor Associado]

“Linha 88: indicar data exata de download da base.” [Revisor 2]

“Linha 119: comentar proporção relativamente alta de ‘indeterminado’ (5,9%). E levar para discussão para ampliar os argumentos.” [Revisor 2]

“Linhas 162-165: esclarecer que o estudo não realizou análise espacial, embora dialogue com literatura que fez.” [Revisor 2]

“• Vulnerabilidade Étnico-Social: ... A discussão ganharia mais relevância se explorasse as barreiras culturais e geográficas específicas que essas populações enfrentam, indo além da simples constatação estatística.” [Revisor 3]

“1. Remover similaridades identificadas no ‘Relatório iThenticate’... revise o texto para assegurar a originalidade...” [Revisor 4]

- Comentários desejáveis (correções menores)

“2- Resumo (objetivo): Utilizar hífen para intervalos de data (ex: 2008-2024). Ajustar as outras menções semelhantes no texto principal e ilustrações;” [Revisor 5]

- Novas recomendações editoriais (adicionais) com base na R1

Resumo

Redação mais direta do método de tendência Trecho atual: “A análise da tendência de aumento ou redução da doença ao longo do tempo foi realizada por meio de testes estatísticos específicos (Mann-Kendall)” Sugerido: “A análise de tendência foi realizada pelo teste Mann-Kendall.”

No trecho atual: “... com uma taxa média de 25,99 casos por 100 mil habitantes.”, esclarecer se é a média das taxas anuais ou uma taxa única do período; alternativamente, reportar taxa inicial e final do período no resumo.

Evitar redundância na conclusão do resumo Trecho atual: “... transmissão ativa e estável ... sem apresentar redução ao longo dos 17 anos...”

Introdução

Na justificativa acrescentada e rever a redação para evitar repetições.

Métodos

Remover referência à figura no delineamento - Trecho atual: “Analisou-se o período de 2008 a 2024 (Figura 1).”

Rever a redação para evitar repetições entre Delineamento/Contexto/Fonte de dados, por exemplo, consolidar descrições institucionais e a referência (11) apenas em “Fonte de dados”.

Conferir e padronizar os nomes das variáveis nos Métodos e tabelas, por exemplo, ‘tipo de entrada’ consta nos Métodos e não localizei na tabela. Inclusive, rever na Tabela o nome das áreas geográficas e manter regiões de saúde ou municípios (para não gerar confusão que trata-se de desagregações distintas.

Justificar sucintamente o uso do Mann-Kendall: Inserir algum comentário sobre aplicabilidade (teste não paramétrico, tendência monótona, robustez a não normalidade/outliers).

Resultados

Reordenar o texto para favorecer a leitura Sugerido: (i) Tabela 1 (seguindo a sequência das variáveis em Métodos); (ii) Figura 1 (comentar apenas a figura/mapa); (iii) Figura 2 (descrever taxas - mínima, máxima, picos/vales - e, ao final, a tendência pelo MK).

Se factível, testar tendência por regiões e sintetizar (inclusive como “dados não apresentados”), indicando se a estabilidade é uniforme; se não for realizado, evitar a formulação vaga da limitação e justificar objetivamente a opção metodológica.

Discussão

Trecho a revisar nas limitações: “Não foi feita uma análise detalhada por município, afim de compreender os padrões de cada um...” (corrigir também ‘a fim’)

Conclusão 

Ajustar a menção à Saúde Única para evitar solução “declaratória” Trecho atual: “... é necessário adotar o conceito de Saúde Única, unindo o tratamento das pessoas ao controle dos insetos e à preservação equilibrada do ambiente...” Sugerido: enfatizar ações intersetoriais e sustentadas alinhadas à abordagem de Saúde Única (vigilância/diagnóstico, manejo clínico, controle vetorial, melhorias ambientais e urbanas), evitando sugerir que a mera “adoção do conceito” resolva o problema.

Permanecemos à disposição para quaisquer esclarecimentos e agradecemos, mais uma vez, pela submissão à RESS.

Atenciosamente,

Max Moura de Oliveira

Editor Associado

Recommendation: Minor revision 

## Comments

Prezados autores,

Após a análise detalhada do histórico e das revisões do manuscrito intitulado “Leishmaniose tegumentar americana no norte de Minas Gerais: análise epidemiológica e série temporal, 2008-2024” (ID: RESS-2025-1212), a versão revisada apresenta avanços significativos em relação às versões anteriores.

O estudo atende, em grande parte, à maioria das recomendações emitidas pelos revisores e pela equipe editorial, demonstrando o esforço dos autores em ajustar o manuscrito para adequar-se às normas e estratégias editoriais da Epidemiologia e Serviços de Saúde (RESS).

No entanto, ainda há pequenos ajustes necessários antes da aceitação final do manuscrito. Essas questões são pontuais e corrigíveis, sem comprometer a validade científica do estudo. Pontos a serem ajustados no manuscrito:

1. Revisar o último parágrafo da introdução, de modo a evitar repetições e garantir que a justificativa culmine de forma clara na apresentação do objetivo do estudo. Sugerimos o seguinte texto como referência, com adequações de estilo e número de palavras para se adequar ao limite da RESS:

“Apesar da relevância epidemiológica da leishmaniose tegumentar americana, ainda há lacunas na compreensão de sua dinâmica regional, especialmente em áreas de alta vulnerabilidade como o Norte de Minas Gerais. Essa região apresenta particularidades socioambientais e epidemiológicas que sustentam a persistência da transmissão, mesmo diante de esforços de controle. Este estudo se diferencia ao realizar uma análise de uma série temporal extensa, possibilitando a identificação de padrões de persistência e tendências de longo prazo. Esses achados são essenciais para subsidiar intervenções estratégicas e direcionadas em saúde pública. Assim, o objetivo deste estudo foi analisar o perfil epidemiológico e a tendência temporal da leishmaniose tegumentar americana na macrorregião Norte de Minas Gerais entre os anos de 2008 e 2024.”

2. Na tabela, sugere-se que a categoria “ignorado/em branco” seja posicionada como última em todas as variáveis. Além disso, é necessário incluir nas limitações como a incompletude dos dados pode impactar a análise epidemiológica e a vigilância.

3. Recomenda-se incluir legenda na Figura 2, para detalhar o que os pontos representam (valores anuais) e o papel da linha (tendência suavizada). Esse ajuste tornará a visualização das figuras mais informativa e autoexplicativa.

Agradecemos o empenho dos autores em aprimorar o manuscrito e reforçamos que estas alterações contribuirão para a qualidade e clareza do estudo, alinhando-se às diretrizes da Epidemiologia e Serviços de Saúde (RESS).

Permanecemos à disposição para quaisquer esclarecimentos ou dúvidas adicionais.

Atenciosamente,

Max Moura de Oliveira

Editor Associado

Epidemiologia e Serviços de Saúde

Recommendation: Minor revision 

